# Induced alternative splicing an opportunity to study PCSK9 protein isoforms at physiologically relevant concentrations

**DOI:** 10.1038/s41598-023-47005-y

**Published:** 2023-11-13

**Authors:** Jessica M. Cale, Kristin A. Ham, Dunhui Li, Craig S. McIntosh, Gerald F. Watts, Steve D. Wilton, May T. Aung-Htut

**Affiliations:** 1 Centre for Molecular Medicine and Innovative Therapeutics, Health Futures Institute, Murdoch University, 90 South Street, Murdoch, WA 6150 Australia; 2 Perron Institute for Neurological and Translational Science, Perth, WA 6009 Australia; 3 School of Medicine, Faculty of Health and Medical Sciences, University of Western Australia, Perth, WA 6009 Australia; 4 Cardiometabolic Clinic, Departments of Cardiology and Internal Medicine, Royal Perth Hospital, Perth, WA 6000 Australia

**Keywords:** Biochemistry, Biological techniques, Molecular biology

## Abstract

Splice modulating antisense oligomers (AOs) are increasingly used to modulate RNA processing. While most are investigated for their use as therapeutics, AOs can also be used for basic research. This study examined their use to investigate internally and terminally truncated proprotein convertase subtilisin/kexin type 9 (PCSK9) protein isoforms. Previous studies have used plasmid or viral-vector-mediated protein overexpression to study different PCSK9 protein isoforms, creating an artificial environment within the cell. Here we designed and tested AOs to remove specific exons that encode for PCSK9 protein domains and produced protein isoforms at more physiologically relevant levels. We evaluated the isoforms’ expression, secretion, and subsequent impact on the low-density lipoprotein (LDL) receptor and its activity in Huh-7 cells. We found that modifying the Cis-His-rich domain by targeting exons 10 or 11 negatively affected LDL receptor activity and hence did not enhance LDL uptake although the levels of LDL receptor were increased. On the other hand, removing the hinge region encoded by exon 8, or a portion of the prodomain encoded by exon 2, have the potential as therapeutics for hypercholesterolemia. Our findings expand the understanding of PCSK9 isoforms and their impact on the LDL receptor and its activity at physiologically relevant concentrations.

## Introduction

Plasmid or viral-vector-mediated protein overexpression has been a valuable tool for researchers investigating the function of wild-type and various protein isoforms^1–5^. However, these systems produce proteins that are generally several-fold higher in abundance than naturally occurring endogenous levels and can induce non-specific interactions with other cellular contents, such as proteins or RNA, leading to inaccurate experimental conclusions. Therefore, we used splice modulating antisense oligonucleotides (AOs) to study various protein isoforms in a more subtle and physiologically relevant way, and at levels typically expressed in cells.

Antisense oligonucleotides are short (15–30 mers) synthetic nucleic acids, chemically modified to resist nuclease degradation and anneal to a reverse complementary sequence through Watson–Crick base pairing. Upon binding to their target sequences and depending on the nature of the oligomer chemistry, AOs can initiate one of several mechanisms: RNase H recruitment; RNA silencing; splicing modulation, or manipulating protein translation^6^. For this study, we focused on AO-mediated exon skipping to produce internally or terminally truncated protein isoforms.

The secreted glycoprotein proprotein convertase subtilisin/kexin type 9 (PCSK9) was selected as our target protein for this study. The role of PCSK9 as a negative regulator for low-density lipoprotein receptor (LDLR) was discovered in patients with loss or gain-of-function mutations^7–10^, and also confirmed in both in vitro and in vivo systems^11–13^. It was observed that viral vector-mediated overexpression of PCSK9 led to a reduction of total LDLR through accelerated lysosomal degradation of the receptor itself without any alteration of *LDLR* RNA synthesis. However, contradicting observations were also reported regarding the additional roles of PCSK9. When HEK293 cells overexpressing PCSK9 were used in a study by Emmer et al.^1^, SURF4 was found to promote PCSK9 secretion. However, Shen et al. showed that knocking down SURF4 increased the endogenous expression and secretion of PCSK9 in two hepatoma-derived cell lines, HepG2 and Huh-7^14^. In addition, Gustafsen et al. observed that SORT1-mediated PCSK9 secretion in primary mouse hepatocytes^2^, while another study did not reveal any significant effect on PCSK9 activities in both Huh-7 cells and *Sort1* knockout mice^15^. These contradicting results are most likely due to the studies being performed in different cell types (cultured versus primary cells) and systems (overexpression versus endogenous expression and in vitro versus in vivo). Furthermore, PCSK9 may play additional cell-specific roles that are yet to be discovered.

The 75 kD PCSK9 protein is a single peptide encoded by 12 exons. It consists of a prodomain (PD), a subtilisin-like catalytic domain, a hinge region, and a C-terminal Cys-His-rich domain (CHRD) that can be further subdivided into C-terminal modules (CM) 1, CM2 and CM3. PCSK9 undergoes an autocatalytic cleavage to remove the PD, which remains associated with the rest of the protein. The roles of wild-type and truncated protein isoforms were studied by others using protein overexpression^3–5^. This allowed us to compare our observations of endogenous levels of PCSK9 isoforms induced by splice modulation to those reported by others using protein overexpression.

We showed that internally and terminally truncated PCSK9 proteins are produced after treating cells with splice modulating AOs that induced targeted exon skipping. We assessed these truncated proteins' expression, secretion, and consequences on LDL uptake. Our observations contrast with those previously reported by others^3–5^ and we also uncovered novel findings. Apart from one study that attempted to develop RNA therapeutics through switching PCSK9 isoforms from full-length to one that is missing exon 8^16^, this is the first study to modulate endogenous levels of various PCSK9 isoforms and investigate their impact on LDLR expression and LDL uptake.

Splice modulating AOs are no doubt applicable as therapeutics, with six currently approved by US Food and Drug Administration, four inducing targeted exon skipping in *DMD* gene transcripts, one promoting exon inclusion in *SMN2* transcripts, and one correcting a unique splicing defect in *CLN7* transcripts^17^. Here we showed their application as laboratory tools that enable the study of protein isoforms at physiologically relevant concentrations, providing better insights.

## Methods

### Antisense oligonucleotide design

In silico analysis of motifs involved in *PCSK9* pre-mRNA transcript splicing was performed using SpliceAid^18^. AOs targeting the predicted splicing enhancer motifs, acceptor and donor splice sites were designed to induce targeted exon skipping. The AOs with 2′-*O*-Me (2′OMe) modified nucleotides on a phosphorothioate backbone (PS) were ordered from ChemGenes Corporation (Massachusetts, USA), and phosphorodiamidate morpholino oligomers (PMOs) were purchased from Gene Tools LLC (Oregon, USA). The sequences of 2′OMe PS AOs designed and tested in this study are listed in Supplementary Table 1 and PMOs in Table 1. The nomenclature of all AOs is as previously described^19^.

**Table 1:** PMO names and sequences used in this study.

Names	Sequences
PCSK9 H2A (− 15 + 10)	TCCACGGATCCTGGCCCCATGCAAG
PCSK9 H8A (+ 72 + 92)	GATGACATCTTTGGCAGAGAA
PCSK9 H8D (+ 10 − 15)	TGCCATCCTGCTTACCTGCCCCATG
PCSK9 H9A (+ 114 + 138)	CGCCCCGCCGCTTCCCACTCCTGGA
PCSK9 H10A (+ 150 + 174)	GAGGACGTGGCCCTGTTGGTGGCAG
PCSK9 H10A (+ 145 + 169)	CGTGGCCCTGTTGGTGGCAGTGGAC
PCSK9 H11A (+ 145 + 169)	GCCGGGATTCCATGCTCCTTGACTT
Gene tools control (GTC)	CCTCCTACCTCAGTTACAATTTATA
Dmd M23D (+ 07 − 18)	GGCCAAACCTCGGCTTACCTGAAAT

### Cell culture and transfection/neon electroporation

Unless otherwise stated, all cell culture reagents, transfection and Neon electroporation reagents were sourced from Thermo Fisher Scientific (Victoria, Australia). Human hepatocellular carcinoma cell line, Huh-7, was sourced from CellBank Australia (New South Wales, Australia) and propagated in Dulbecco’s modified Eagle’s media (DMEM) supplemented with 10% fetal bovine serum (FBS; Serana, Western Australia, Australia) in 75 cm^2^ flasks at 37 °C in a 5% CO_2_ incubator.

Huh-7 cells were seeded at a density of 60,000 per well in a 24-well plate one day before transfection with various concentrations of 2′OMe PS AOs using Lipofectamine 3000 transfection reagent (3 µl/ml) according to the manufacturer’s instructions. All 2′OMe PS AO transfections were performed in OptiMEM, and transfected cells were incubated for 24 h.

For PMO delivery, Neon electroporation was performed. Approximately 300,000 Huh-7 cells were collected, washed once with PBS, and resuspended in 10 µl of Resuspension Buffer R/PMO combination according to the manufacturer’s instructions. Electroporation was performed at 1300 V, with one pulse for 30 ms. Cells were plated in a single well of a 12-well plate in DMEM supplemented with 5% FCS for three days before reseeding approximately 50,000 cells on 15 mm round coverslips in DMEM supplemented with 2% FCS for immunolabelling of LDLR. The coverslips were collected the following day (day 4). The remaining cells were divided such that 20% were used for *PCSK9* transcript analysis, and 80% were set aside for PCSK9 expression via western blotting. The cell culture media was collected and centrifuged at 3000 rpm to sediment any cells/debris, and the supernatant was collected to measure PCSK9 protein secretion. For analysis by flow cytometry, the Neon electroporation experiment was repeated, and the cells were plated in a single well of a 12-well plate in DMEM supplemented with 5% FCS for three days before the media was replaced with DMEM supplemented with 2% FCS for one day. For nonsense mediated decay inhibition, cells were incubated with 50 µg/ml Cycloheximide (CHX) for 4 or 24 h prior to collection.

Untreated and Gene Tools control (GTC) treated samples were included in all experiments. An untreated zap control with no AO was included in the Neon electroporation experiments. Dmd M23D, an AO targeting murine dystrophin mRNA, was included as an unrelated control for the flow cytometry analysis. After treatments, cell viability was analysed using Incucyte® SX5 confluence software.

### RNA extraction, cDNA synthesis and PCR

Total RNA was extracted using the MagMax™ 96 total RNA isolation kit (AM1830; Thermo Fisher Scientific), according to the manufacturer’s instructions. The SuperScript™ IV First-Strand Synthesis System (Thermo Fisher Scientific) was used for cDNA synthesis. Five microliters of the total RNA was used for 10 µl cDNA synthesis following the manufacturer’s instructions and the thermocycling conditions: 23 °C for 10 min, 50 °C for 10 min and 80 °C for 10 min. Approximately 50 ng of cDNA was used as a template for PCR amplification using the TaKaRa LA Taq® DNA Polymerase with GC II buffer system (Takara Bio, California, USA). Superscript III One-Step RT-PCR system (Thermo Fisher Scientific) was used to analyse the housekeeping *SMN, TBP* and cycloheximide positive control *HNRNPD* transcripts. Approximately 50 ng of total RNA was used as a template. Primers (Integrated DNA Technologies, Iowa, USA) and PCR conditions are listed in Table 2. Exon skipping efficiencies and percentage knock-down of *PCSK9* transcript were calculated after densitometric analysis of the full-length *PCSK9* and housekeeping *SMN* and *TBP* transcripts. The percentage of various *PCSK9* transcript isoforms was calculated after normalising against the housekeeping transcripts and compared to the untreated sample. The RT-qPCR reactions were performed using the TaqMan™ Fast Advanced Master Mix (Thermo Fisher Scientific), according to the manufacturer’s instructions. The reactions were performed in triplicates using a CFX384 Touch Real-Time PCR detection system (Bio-Rad Laboratories Pty., Ltd., New South Wales, Australia), and *PCSK9* (Integrated DNA Technologies) transcript expression relating to the reference transcript *TBP* (Thermo Fisher Scientific) was calculated. The expression assays are listed in Table 2. Threshold cycle (Ct) values were determined using the CFX Maestro Software 2.3 (Bio-Rad Laboratories). Relative expression of *PCSK9* to *TBP* mRNA was calculated using the comparative Ct or 2^−∆∆Ct^ method^20^ and presented as a fold-change compared to the untreated sample.

**Table 2:** Primer names and sequences used in this study.

Names*	Sequences	PCR conditions
PCSK9_ex1F	GAGGAGCTGGTGCTAGCCTTG	94 °C 1 min 28 cycles of 94 °C 30 s 60 °C 30 s 72 °C 1 min 30 s
PCSK9_ex7R	GGCAAAGAGGTCCACACAGC	
PCSK9_ex6F	GACGATGCCTGCCTCTACTC	
PCSK9_ex12R	GTGCTGCCTGTAGTGCTGA	
SMN_F	AGGTCTCCTGGAAATAAATCAG	55 °C 30 min 94 °C 2 min 25 cycles of 94 °C 30 s 56 °C 30 s 68 °C 1 min
SMN_R	TGGTGTCATTTAGTGCTGCTCT	
TBP_ex2F	AGCGCAAGGGTTTCTGGTTT	55 °C 30 min 94 °C 2 min 24 cycles of 94 °C 30 s 58 °C 30 s 68 °C 1 min
TBP_ex3R	GGAGTCATGGGGGAGGGATA	
HNRNPD_ex1F	TTGACGCCAGTAAGAACGAG	55 °C 30 min 94 °C 2 min 27 cycles of 94 °C 30 s 58 °C 30 s 68 °C 1 min
HNRNPD_in8R	ATACTGCTTCACCACCAAACG	
PCSK9 TaqMan expression assay	Hs.PT.58.20317141	95 °C 20 s 40 cycles of 95 °C 1 s 60 °C 20 s
TBP TaqMan expression assay	Hs00427620_m1	

*PCSK9 transcript ID; NM_174936.4, *SMN* transcript ID; NM_017411.4, *TBP* transcript ID; NM_003194.5. *HNRNPD* transcript ID; NM_031370.3.

### Western blot

Western blot analysis of PCSK9 protein secreted into the growth media was performed on approximately 35 µg of total supernatant protein as measured by Pierce BCA Protein Assay Kit (Thermo Fisher Scientific). Protein loading was assessed by either Ponceau S staining or No-Stain Protein Labeling Reagent (Thermo Fisher Scientific) prior to blocking and antibody probing. Western blot analysis of PCSK9 and housekeeping proteins beta-tubulin and beta-actin were performed on approximately 20 µg of total cellular protein using rabbit polyclonal anti-PCSK9 antibody (cat. no. ABS1006; Sigma-Aldrich, New South Wales, Australia) at a 1:1000 dilution, mouse monoclonal anti-beta-tubulin (cat. no. AB_2315513; Developmental Studies Hybridoma Bank, Iowa, USA) at 1:5000 and mouse monoclonal anti-beta-actin (cat. no. A5441; Sigma-Aldrich) at 1:100,000. Primary antibodies were incubated overnight at 4 °C with gentle agitation. Goat anti-rabbit immunoglobulins/HRP (cat. no. P0448; Dako, Victoria, Australia) was used to visualise PCSK9 at a dilution of 1:10,000 after incubation for one hour at room temperature and visualised using Crescendo western HRP substrate. Anti-mouse secondary AP substrate from WesternBreeze chromogenic kit (Thermo Fisher Scientific) was used to visualise beta-tubulin and beta-actin. Blot images were captured using the Fusion FX system (Vilber Lourmat, Marne-la-Vallee, France) and quantified using Image J^21^ (Rasband, W.S., ImageJ, U.S. National Institutes of Health, Maryland, USA).

### Immunocytochemistry

The cells seeded on coverslips were fixed in ice-cold acetone: methanol (1:1) for 4 min and stored at – 80 °C until immunolabelling was carried out. Coverslips were rinsed with TBST (0.2% Triton) before blocking with 10% filtered normal goat serum diluted in TBST for 30 min at room temperature. Coverslips were subsequently incubated with anti-LDLR antibody (cat. no. sc18823; Santa Cruz, Texas, USA) at a dilution of 1:200 or anti-LAMP1 antibody (cat. no. D2D11, Cell Signaling Technology, Massachusetts, USA) at a dilution of 1:100 in 1% filtered goat serum diluted in TBST for 1 h at room temperature. The excess antibody solution was removed by washing with TBST three times for 5 min each. The Alexa 568 labelled goat anti-mouse secondary antibody (1:400 dilution in 1% filtered goat serum diluted in TBST) (cat. no. A-11011; Thermo Fisher Scientific) was applied to the coverslips for 1 h at room temperature, and the washing steps were repeated. Anti-PCSK9 antibody was then applied at a dilution of 1:250 in 1% filtered goat serum diluted in TBST for another hour, washed three times with TBST and visualised using Alexa 488 labelled goat anti-rabbit secondary antibody (1:400 dilution in 1% filtered goat serum diluted in TBST) (cat. no. A-11008; Thermo Fisher Scientific). Nuclei were stained with Hoechst at a dilution of 1:160 for 3 min in the last wash, and coverslips were mounted onto microscope slides using ProLong Gold Antifade Mountant (Thermo Fisher Scientific). Images were captured using a Nikon Eclipse 80i microscope or Echo Revolve and analysed by NIS-Elements software. For the analysis of LDLR expression, at least 800 cells were analysed for the fluorescent signals, and the operator was blinded.

### LDL-C uptake measurement by flow cytometry

LDL uptake was determined using an LDL Uptake Assay Kit for flow cytometry (cat. no. ab236208; Abcam, Victoria, Australia). On day 4, the culture medium was replaced with 400 µl/well LDL-DyLight™ 488 assay reagent prepared in serum-free DMEM (1:500) and filtered. The cells were incubated at 37 °C in the dark for 3 h before collecting via trypsinisation and resuspended in 200 µl of cold PBS. Data was acquired on a Gallios Flow Cytometer (Beckman Coulter, New South Wales, Australia) and analysed with FlowJo software (TreeStar, Oregon, USA).

## Results

### Analysis of *PCSK9* transcript

**Figure 1:**
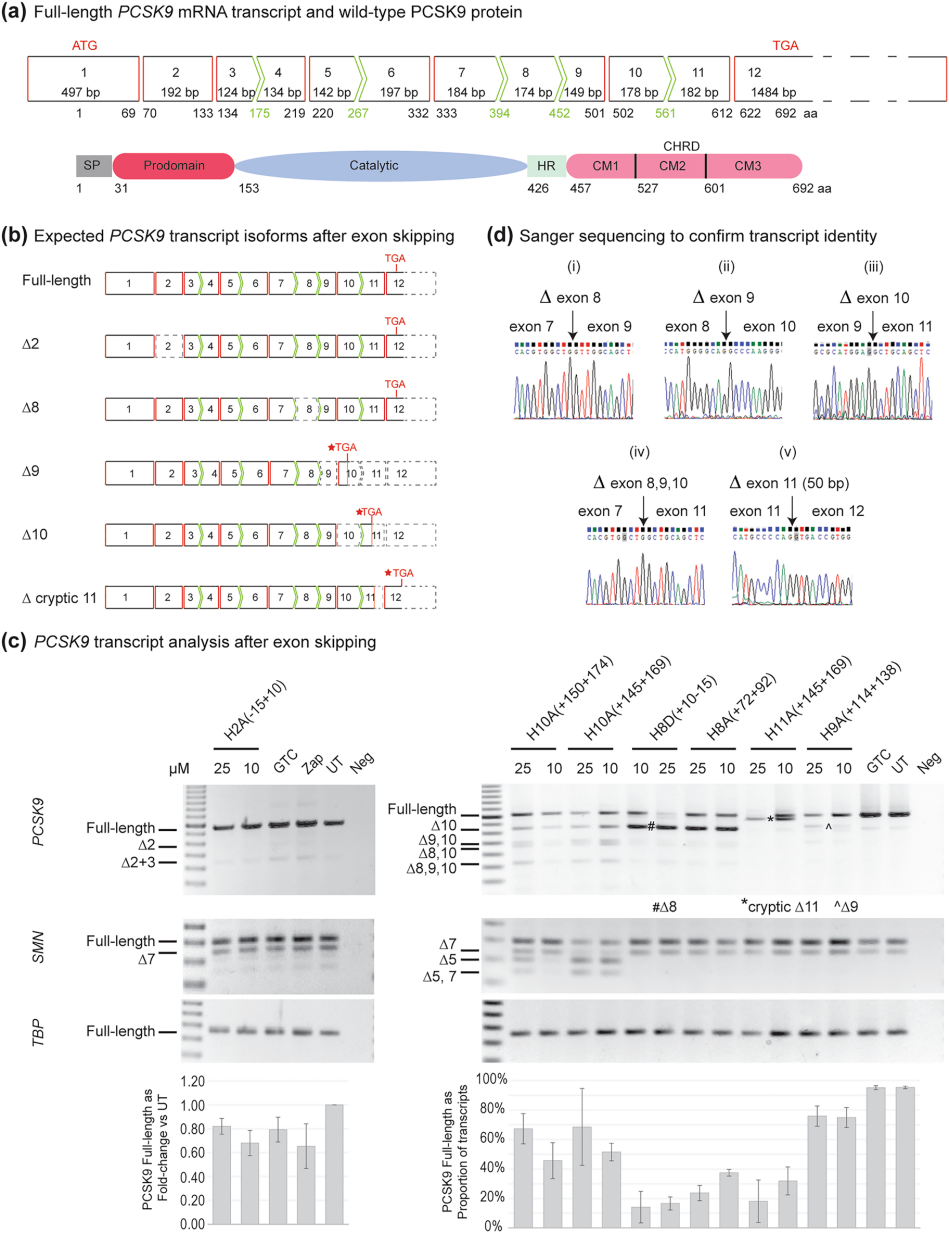
Phosphorodiamidate morpholino oligomer (PMO)-mediated exon skipping of proprotein convertase subtilisin kexin 9 (*PCSK9*) gene transcript. (**a**) Schematic representation of the wild-type *PCSK9* mRNA. Exons are represented as boxes with straight red lines or green chevrons to indicate in-frame and out-of-frame exons, respectively. The exon numbers are shown as Arabic numbers, and each exon's size is also indicated as base pair (bp). The PCSK9 domains encoded by the exons are shown below. PCSK9 protein consists of a signal peptide, prodomain, catalytic domain, hinge region (HR) and C-terminal Cys-His-rich domain (CHRD) which is further subdivided into C-terminal module (CM) 1, CM2 and CM3. The amino acid numbers corresponding to junctions between domains and exons are also shown below. (**b**) Predicted STOP codon locations in *PCSK9* transcripts after exon skipping. New STOP codon locations are indicated with red stars. The grey dotted lines indicate an untranslated region. (**c**) Assessment of exon skipping from *PCSK9* transcripts in Huh-7 cells after treatment with PMO for three days using RT-PCR. Both survival motor neuron (*SMN*) and TATA-box binding protein (*TBP*) transcripts were amplified as internal controls for RNA quality. Semi-quantitative densitometric analysis is displayed as fold-change in full-length compared to the UT for the exon 2 targeting PMO and *PCSK9* full-length as proportion of total PCR products for the remaining targets. Error bars represent the standard error measurement calculated across at least three replicates. Dated replicate experiments are shown in Supplementary Fig. 2. Δ; removal of an exon, GTC; sample treated with Gene Tools control PMO, UT; untreated sample, Neg; RT-PCR without template added. (**d**) Sanger sequencing results to confirm exon skipping. The gel images were cropped for presentation. Full-length original gel images are shown in Supplementary Fig. 3.

Analysis of the *PCSK9* transcript (NM_174936.4), exon composition and the protein domains (Fig. 1a) prompted the hypothesis that internally or prematurely truncated PCSK9 protein isoforms could be induced using AOs designed for targeted exon skipping. The *PCSK9* gene has 12 exons, and only exons 2 and 8 are in-frame, encoding the prodomain and hinge region, respectively. Eight other exons (3, 4, 5, 6, 7, 9, 10 and 11) are out-of-frame, and hence removing any of these individual exons would lead to a shift in the reading frame and may render the induced mRNA transcript susceptible to degradation through nonsense-mediated decay (NMD). However, our previous study showed that some transcripts escaped NMD when the premature stop codon was shifted to the penultimate exon after induction of targeted exon skipping^22^. Hence, in addition to the two in-frame exons, exons 2 and 8, we designed exon skipping AOs targeting exons 9, 10, or 11 to assess whether terminally truncated PCSK9 protein isoforms are produced.

The removal of exons 2 or 8 should produce internally truncated PCSK9 proteins (∆2 or ∆8), missing most of the prodomain (64 amino acids) encoded by exon 2 or the hinge region (58 amino acids) by exon 8, respectively. The canonical stop codon in exon 12 should be maintained in both ∆2 and ∆8 isoforms (Fig. 1b, Supplementary information). However, skipping exons 9 (∆9) or 10 (∆10) should cause a reading frameshift and introduce a premature stop codon in exons 10 and 11, respectively (Fig. 1b). Removing exon 11 (∆11) will also disrupt the reading frame and introduce a premature termination codon 160 nucleotides before the canonical stop codon in exon 12 (Fig. 1b).

### Exon skipping efficacies

In our initial screen using 2′OMe PS AOs, we observed highly variable exon skipping efficacies (Supplementary Fig. 1) ranging from 3 to 80% compared to the negative control AO recommended by Gene Tools LLC (Gene Tools control: GTC). Of these, we selected one sequence targeting exon 2, two each for exon 8 (one is a previously reported sequence^16^) and exon 10, and one each for exons 9 and 11. One AO targeting exon 11 was also selected for further analysis after it was found to activate a donor cryptic splice site within that exon, removing the last 50 nucleotides and disrupting the reading frame. These particular AOs were purchased as PMOs for subsequent functional studies. We previously found the 2′OMe PS AOs can be toxic, generate substantial off-target effects^23^ and are not ideally suited for assessing functional protein in treated cells^24^. Apart from the cells treated with exon 10 targeting PMOs (60% cell death), no significant cell death was observed for other treatments (Supplementary Fig. 4).

Comparison of *PCSK9* exon skipping after 2′OMe PS AO (Supplementary Fig. 1) and PMO (Fig. 1c) treatments showed that blocks of exons are skipped in 2′OMe PS AOs treated samples, while PMO treatments preferentially induced single exon skipping, with the exception of exon 10. In Huh-7 cells treated with 2′OMe PS AOs targeting exon 2, both exon 2 and 3 were preferentially removed from the *PCSK9* transcript (confirmed by Sanger sequencing Supplementary Fig. 1), resulting in a frame-shifted transcript (Fig. 1c). On the other hand, these transcripts with exons 2 and 3 removed were hardly visible in the samples treated with the exon 2 targeting PMO. Reduction in the full-length *PCSK9* was confirmed by RT-qPCR (Supplementary Fig. 1). All exon skipping events were confirmed by Sanger sequencing (Fig. 1d).

Interestingly, only cryptic exon 11 splicing was detected after treating Huh-7 cells with exon 11 PMO (Fig. 1c), whereas the same 2′OMe PS AO sequence induced both cryptic and entire exon 11 skipping (Supplementary Fig. 1). Exon 8 skipping was more efficient with H8D(+ 10 − 15) than H8A(+ 72 + 92), a shorter 20 mer, leaving no detectable full-length *PCSK9*. Similarly, the PMO targeting exon 11 also caused a complete knock-down of full-length *PCSK9*. Generally, all PMO sequences caused a reduction of full-length *PCSK9* transcript. Both PMOs targeting exon 10 affected survival motor neuron (*SMN*) splicing, resulting in exon 5 skipping and low levels of natural exon 7 skipping, indicating cellular stress^25^. However, we confirmed that exon 5 skipping did not affect the formation of “gems”, a functional assessment for SMN protein (Supplementary Fig. 5) when SMN protein was detected by immunolabelling.

### PCSK9 protein isoforms expression and cellular distribution

**Figure 2:**
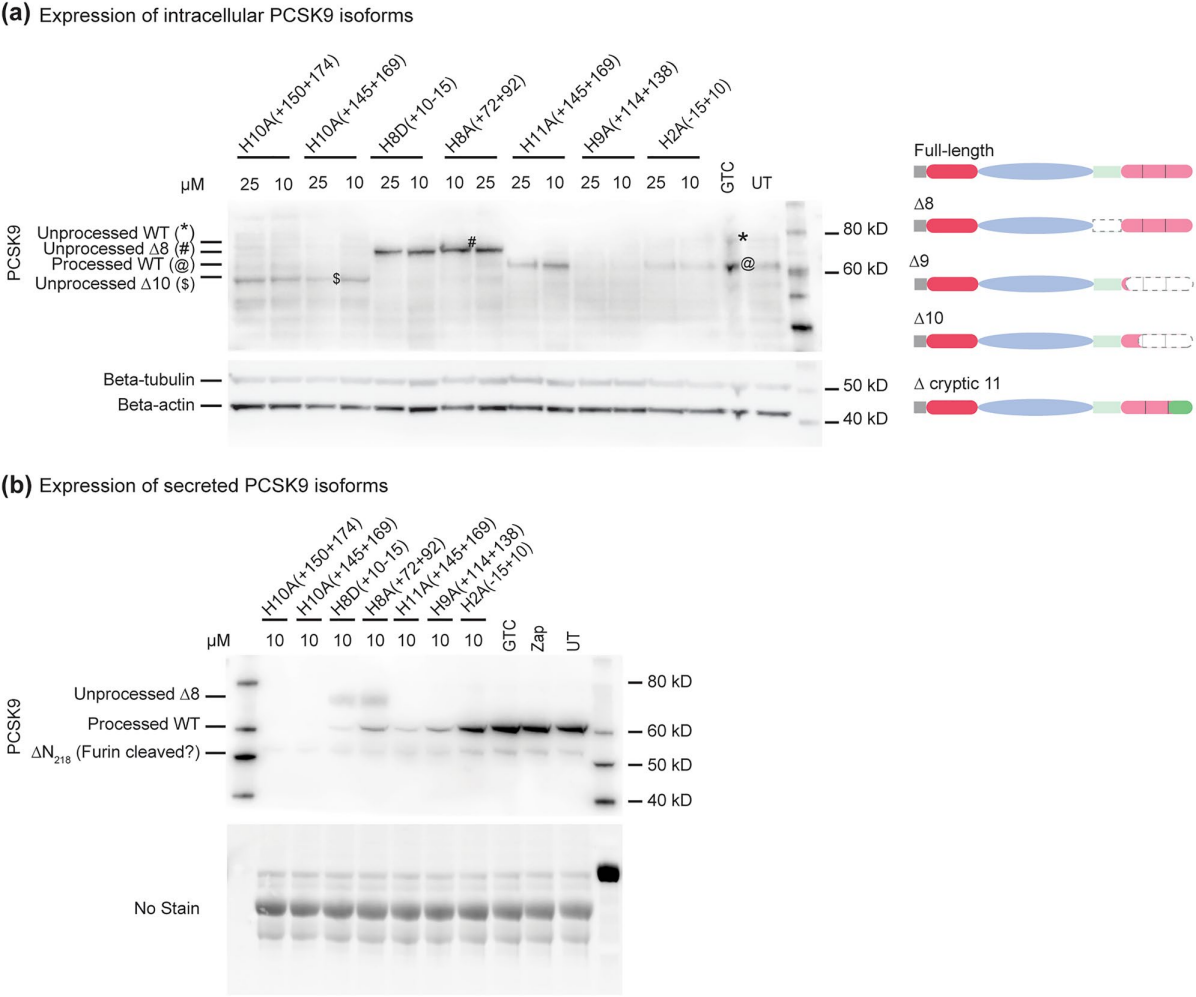
PCSK9 protein isoform expression was assessed in Huh-7 cells after treating with PMOs for 3 days. Western blot analysis of (**a**) intracellular PCSK9 protein isoforms and (**b**) secreted PCSK9 isoforms after targeted exon skipping. Dated replicate experiments are shown in Supplementary Fig. 6. The schematics of the predicted PCSK9 isoforms after exon skipping are shown on the right. The domain(s) predicted to be absent are shown in grey dotted lines. The location of new amino acids introduced into PCSK9 are shown in dark green. WT; wild-type, GTC; sample treated with Gene Tools control PMO, UT1; sample underwent Neon electroporation without any PMO, UT2; untreated sample. The images were cropped for presentation. Full-length original images are shown in Supplementary Fig. 3.

After the successful induction of exon skipping, we analysed the intracellular levels of PCSK9 protein isoforms (Fig. 2a). The processed 62 kD PCSK9 is present in untreated, and samples treated with GTC, exon 2 or 11 targeting PMOs. The molecular weight of the expected PCSK9 isoform after exon 2 skipping or cryptic exon 11 processing is similar to that of the wild-type PCSK9 (Table 3). Hence, we were unable to confirm whether the observed PCSK9 is the wild-type or the AO-induced isoforms. However a reduction in PCSK9 after treatment with the exon 2 targeting PMO was observed indicating the PMO may cause an unidentified splicing product that could lead to protein knockdown. This requires further investigation. Dual exon skipping (exon 2 and 3) is a frame-shift transcript and would likely lead to NMD and hence protein knock-down; however, inhibition of nonsense mediated decay showed no increase in the exon 2 and 3 skipped products (Supplementary Fig. 2).

**Table 3:** Predicted PCSK9 protein truncations and processing.

Target exon	Residues removed*	Predicted MW (unprocessed, processed)
Full-length	N/A	75 kD, 62 kD
Exon 2	70–133	67 kD, 62 kD
Exon 8	394–452	68 kD, 55 kD
Exon 9	452–692 (+ 16)	51 kD, 38 kD
Exon 10	502–692 (+ 18)	57 kD, 44 kD
Exon 11	605–692 (+ 87)	75 kD, 62 kD

*New residues are indicated in parentheses.

*N/A* not applicable.

All three out-of-frame exon skipping events (∆9/∆10/∆11) led to reduction of total PCSK9 protein expression (Fig. 2a and b). We also successfully induced a truncated 68 kD ∆8 PCSK9 protein after exon 8 exclusion, while exon 10 skipping led to the production of a 57 kD ∆10 PCSK9 isoform. Based on the predicted versus observed molecular weights (Table 3), these novel PCSK9 isoforms missing the hinge region (∆8) or CM2-3 domain (∆10) are likely to be unprocessed. The level of ∆8 PCSK9 isoform was higher than those observed for the wild-type PCSK9 in GTC and untreated samples. We cannot account for the consistent loss of processed WT PCSK9 observed in the cell lysate of the samples treated with the exon 10 PMOs (Fig. 2a) despite the main transcript being the full-length (Fig. 1c). In our experience, RNA expression does not always equivalate “one to one” to protein expression. In addition, the exon 10 AOs are toxic to the cells, as shown by both cell counts (Supplementary Fig. 4) and the *SMN* RT-PCR showing exon 5 and 7 skipping (Fig. 1c). Perhaps the isoforms induced by treating the cells with exon 10 PMOs have a toxic gain of function.

In addition, we assessed PCSK9 secretion for all treated and untreated samples (Fig. 2b). In both untreated and GTC treated samples, PCSK9 protein is mainly secreted. Generally, we observed lower levels of PCSK9 secretion for all PMO treated samples. Interestingly, an increased proportion of a protein band around 50 kD, possibly furin cleaved PCSK9-ΔN_218_, was observed for those cells treated with exon 8, 10, 11 and exon 9 (only at high concentration) targeting PMOs in some experiments. For the samples treated with exon 10 targeting PMOs, although the wild-type processed PCSK9 was barely visible in cell lysate, low levels were found in the supernatant at 10 µM in some experiments. Unlike what had been previously reported^26^, the unprocessed ∆8 PCSK9 missing the hinge region was found to be secreted. We also analysed the cellular distribution of PCSK9 isoforms using immunocytochemistry and did not observe any alteration (Supplementary Fig. 7).

### LDLR expression and LDL uptake in cells expressing PCSK9 protein isoforms.

**Figure 3:**
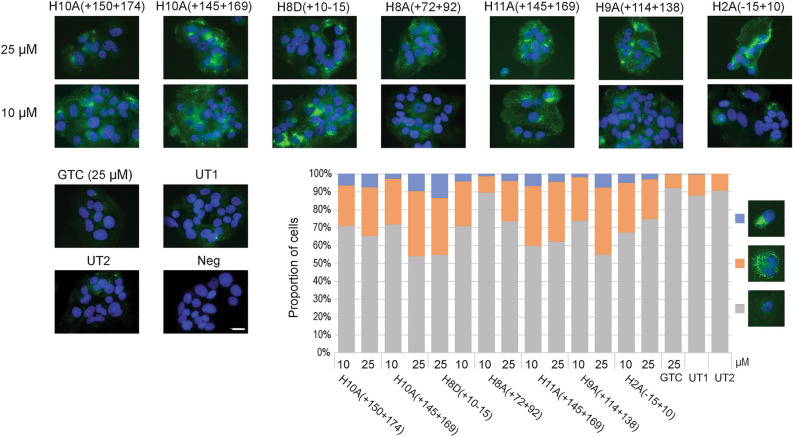
Analysis of low-density lipoprotein receptor (LDLR) expression in Huh-7 cells after PMO treatment for 4 days. GTC; sample treated with Gene Tools control PMO, UT1; sample underwent Neon electroporation without any PMO, UT2; untreated sample. Scale bar 20 µm. Immunostaining of LDLR protein expression (green); nuclei (blue).

Next, we examined the effects of the PMO induced PCSK9 isoforms on the expression and activity of LDLR since PCSK9 has been shown to negatively regulate LDLR expression and activity. Generally, all PMO treatments enhanced LDLR expression in a dose-dependent manner compared to GTC treated and untreated samples (Fig. 3). At 25 µM concentrations, all PMO treatments led to 30–40% of Huh-7 cells saturated with LDLR, which is four-fold higher than those observed in GTC and untreated samples. These LDLR do not co-localise with lysosome-associated membrane protein 1 (LAMP-1) when both proteins were analysed through immunolabelling (Supplementary Fig. 8).

**Figure 4:**
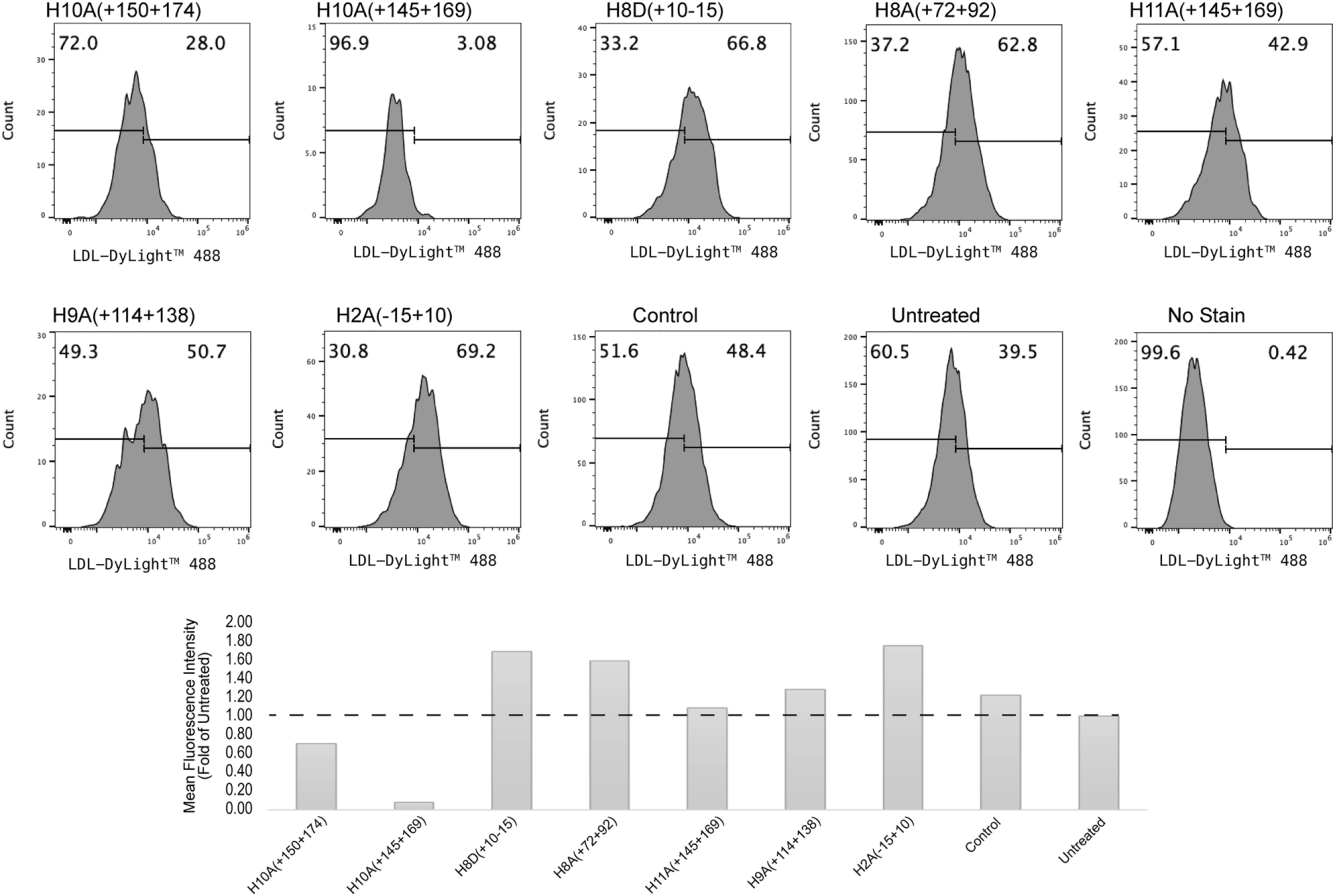
Flow cytometric analysis of low-density lipoprotein (LDL) uptake in Huh-7 cells after 10 µM PMO treatment for 4 days. Fluorescence frequency distribution plot of samples (top) and the mean fluorescence intensity fold change compared to untreated (bottom). Control; sample treated with Dmd M23D(+ 07 − 18) PMO. The PMO identities are shown above the histograms and below the bars. The no stain sample indicates cells not treated with LDL uptake assay.

Next, we assessed the LDL uptake in Huh-7 cells treated with PMOs (Fig. 4). We included the siRNA control to ensure optimal LDL uptake assay conditions (Supplementary Fig. 9). Of all PMO treated Huh-7 cells, only exon 2 and 8 PMO treated samples showed an increase in uptake of LDL despite the evidence of an increase in the expression of LDLR (correlated with reduced PCSK9 expression) for all PMO treatments via immunolabelling (Fig. 3). These results indicated that the activity of PCSK9 was compromised when exon 2 or 8 was removed, and hence the LDLR expression was increased, and the LDL uptake was enhanced. However, the PCSK9 isoforms (∆10 or ∆11) with altered or truncated CHRD still negatively affect LDLR activity and hence the LDL uptake was not increased. Interestingly, although there was a reduced expression of PCSK9 and increased levels of LDLR after exon 9 skipping, LDL uptake remained the same. It is possible that the ∆9 isoform was not detected due to the antibody not recognising the altered PCSK9 isoform.

## Discussion

As evidenced by the recent FDA approvals of splice modulating AOs, splice modulation is a powerful strategy for therapeutic application. Here we showed that these AOs could also be used as a laboratory tool to investigate the potential roles of protein isoforms at physiologically relevant concentrations. We have selected PCSK9 expression to induce various isoforms using steric blocking AOs for targeted splice modulation, in particular targeted exon skipping since the activity of PCSK9 can be indirectly assessed via LDLR activity, and studies on PCSK9 protein isoforms using protein overexpression are available for comparison.

The proposed study on protein isoforms using splice modulation is most suitable for genes where alternative transcript isoforms are naturally present. Previously, we reported AO-mediated exon skipping studies for several genes^27–30^. We consistently observed efficient exon skipping for alternatively spliced exons and variable exon skipping efficiencies for other exons. This observation is also evident in this study. Robust exon skipping was achieved for exon 8, an alternative spliced exon. In addition, inducing exon 2 skipping also resulted in exon 2 and 3 removal from the *PCSK9* mRNA, as this isoform is already present in the untreated cells.

We have successfully modified PCSK9 protein expression after targeted exon skipping, and these isoforms remained intracellular except the Δ8 isoform. Hence, any impact observed for LDLR activity by these isoforms was mainly via intracellular interactions. Exon 8 or 10 skipping resulted in the internal and terminal deletion of a significant portion of PCSK9 protein, respectively. These proteins were evident in western blot analysis. Identification of PCSK9 protein isoforms Δ2, Δ9 or Δ11 faced challenges as the sizes of new isoforms are similar to that of the wild-type. However, the downstream assessment of the LDLR activity on LDL uptake indicated that by removing exon 9 or 11, we produced PCSK9 isoforms that tightly bind to LDLR, preventing it from going through the degradation process and thus hindering LDL uptake. Although we could barely detect the PCSK9 protein after exon 9 skipping, we believe this could be due to the antibody not recognising the new isoform rather than the reduction in the level of PCSK9; since knocking down PCSK9 expression is well documented to enhance LDL uptake, which we did not observe after exon 9 skipping. Inducing exon 2 skipping may either produce an internally truncated protein, which has lost its dominant negative impact on LDLR or result in a slight reduction of PCSK9 protein possibly through an unidentified splicing product.

Our results were inconsistent with a previous report that found the hinge region is important for PCSK9 secretion^13^, as we detected a PCSK9 isoform lacking the entire hinge region (∆8) in proteins derived from the supernatant. In addition, the secreted ∆8 PCSK9 isoform has lost some or all negative regulatory impacts on LDLR receptors, resulting in an increased number of cells with LDLR. Removal of the entire C-terminal or the CM2 and CM3 (amino acids 534 to 692), similar to ∆10 in this study, did not affect PCSK9 secretion when these isoforms were overexpressed in HEK293T cells and HepG2 cells treated with short hairpin RNA targeting *PCSK9*^4,5^. However, the ∆10 PCSK9 isoform appeared to remain intracellular in our study.

The discrepancies observed between our study and others could be due to differences in the cellular systems employed. We could express specific truncated PCSK9 isoforms at physiological or normal endogenous levels. At the same time, other studies utilised overexpression of PCSK9 isoforms, which would be several-fold higher than the levels expected to have been present in Huh-7 cells. This artificial overexpression system could dramatically affect protein turnover and processing, as evidenced by approximately 50% of PCSK9 being unprocessed in such systems^4,5^. On the other hand, in our study, when endogenous expression of PCSK9 was analysed in Huh-7 cells, we mainly observed the processed form, and the majority was secreted. Additionally, non-specific protein interactions could be possible in the cells when PCSK9 is vastly overexpressed beyond normal levels.

One potential drawback of using splice modulating AOs is that the strategy is limited to genes where in-frame exons precisely encode the protein domains, and one must be aware that introducing new amino acids, such as those expected for ∆10 and ∆11 PCSK9 isoforms, may interfere with normal protein folding and consequently affect function. However, we are confident that the unprocessed ∆8 PCSK9 is secreted, which raises questions about previous reports^3^. It should also be noted that the significant size difference between the induced protein isoforms and the wild-type protein helps confirm the production of new isoforms.

This is the first study to explore the suitability of splice modulating AOs for investigating the function of protein isoforms at what could be expected endogenous or normal levels, and this may account for some discrepancies observed in previous reports. While there is no doubt that the splice intervention methodology has limitations, it still represents a more natural environment. Hence, we propose future studies using the splice modulation strategy to confirm the previously reported function and regulation of applicable protein targets. We have identified PCSK9 isoforms with an altered or deleted CHRD to have a dominant negative effect on LDLR, preventing LDL uptake. The PMOs targeting exons 2 and 8 identified in this study have the potential for the development of new therapeutics for regulating PCSK9 to treat hypercholesterolemia^31,32^. Further studies on refining these PMOs and in vivo assessments on their potential for reducing cholesterol could lead to new therapies for patients not compatible with or intolerant of existing lipid-lowering regimes.

### Supplementary Information

Supplementary Information.

## Data Availability

All data generated or analysed during this study are included in this published article (and its Supplementary Information file). “The datasets generated and/or analysed during the current study are available in the GeneBank repository, GenBank accession numbers OR147794-OR147799.
