# Supplementary material for: Induced alternative splicing an opportunity to study PCSK9 protein isoforms at physiologically relevant concentrations
Source: Sci Rep. 2023 Nov 13;13:19725. doi: 10.1038/s41598-023-47005-y (PMC10643364; doi:10.1038/s41598-023-47005-y)
Supplement: Supplementary file 1 — Supplementary Information. [file 41598_2023_47005_MOESM1_ESM.pdf]

Supplementary information

Predicted STOP codon locations in *PCSK9*

TGA canonical

TGA new

Full-length *PCSK9*

Exon 12

GTGACCGTGGCCTGCGAGGAGGGCTGGACCCTGACTGGCTGCAGTGCCCTCCCTGGGACC

TCCCACGTCCTGGGGGCCTACGCCGTAGACAACACGTGTGTAGTCAGGAGCCGGGACGTC

AGCACTACAGGCAGCACCAGCGAAGGGGCCGTGACAGCCGTTGCCATCTGCTGCCGGAGC

CGGCACCTGGCGCAGGCCTCCCAGGAGCTCCAGTGACAGCCCCATCCCAGGATGGGTGTC

Exclusion of exon 2

Exon 12

GTGACCGTGGCCTGCGAGGAGGGCTGGACCCTGACTGGCTGCAGTGCCCTCCCTGGGACC

TCCCACGTCCTGGGGGCCTACGCCGTAGACAACACGTGTGTAGTCAGGAGCCGGGACGTC

AGCACTACAGGCAGCACCAGCGAAGGGGCCGTGACAGCCGTTGCCATCTGCTGCCGGAGC

CGGCACCTGGCGCAGGCCTCCCAGGAGCTCCAGTGACAGCCCCATCCCAGGATGGGTGTC

Exclusion of exon 8

Exon 12

GTGACCGTGGCCTGCGAGGAGGGCTGGACCCTGACTGGCTGCAGTGCCCTCCCTGGGACC

TCCCACGTCCTGGGGGCCTACGCCGTAGACAACACGTGTGTAGTCAGGAGCCGGGACGTC

AGCACTACAGGCAGCACCAGCGAAGGGGCCGTGACAGCCGTTGCCATCTGCTGCCGGAGC

CGGCACCTGGCGCAGGCCTCCCAGGAGCTCCAGTGACAGCCCCATCCCAGGATGGGTGTC

Exclusion of exon 9

Exon 10

GCCCAAGGGGGCAAGCTGGTCTGCCGGGCCCACAACGCTTTTGGGGGTGAGGGTGTCTAC

GCCATTGCCAGGTGCTGCCTGCTACCCCAGGCCAACTGCAGCGTCCACACAGCTCCACCA

GCTGAGGCCAGCATGGGGACCCGTGTCCACTGCCACCAACAGGGCCACGTCCTCACAG

Exclusion of exon 10

Exon 11

GCTGCAGCTCCCACTGGGAGGTGGAGGACCTTGGCACCCACAAGCCGCCTGTGCTGAGGC

CACGAGGTCAGCCCAACCAGTGCGTGGGCCACAGGGAGGCCAGCATCCACGCTTCCTGCT

GCCATGCCCCAGGTCTGGAATGCAAAGTCAAGGAGCATGGAATCCCGGCCCCTCAGGAGC

AG

Exclusion of exon 11 (cryptic splicing)

Exon 11

GCTGCAGCTCCCACTGGGAGGTGGAGGACCTTGGCACCCACAAGCCGCCTGTGCTGAGGC

CACGAGGTCAGCCCAACCAGTGCGTGGGCCACAGGGAGGCCAGCATCCACGCTTCCTGCT

GCCATGCCCCAG~~GTCTGGAATGCAAAGTCAAGGAGCATGGAATCCCGGCCCCTCAGGAGC~~

~~AG~~

Exon 12

GTGACCGTGGCCTGCGAGGAGGGCTGGACCCTGACTGGCTGCAGTGCCCTCCCTGGGACC

TCCCACGTCCTGGGGGCCTACGCCGTAGACAACACGTGTGTAGTCAGGAGCCGGGACGTC

AGCACTACAGGCAGCACCAGCGAAGGGGCCGTGACAGCCGTTGCCATCTGCTGCCGGAGC

CGGCACCTGGCGCAGGCCTCCCAGGAGCTCCAGTGACAGCCCCATCCCAGGATGGGTGTC

TGGGGAGGGTCAAGGGCTGGGGCTGAGCTTTAAAATGGTTCCGACTTGTCCCTCTCTCAG

CCCTCCATGGCCTGGCACGAGGGGATGGGGATGCTTCCGCCTTTCCGGGGCTGCTGGCCT

GGCCCTTGAGTGGGGCAGCCTCCTTGCCTGGAACTCACTCACTCTGGGTGCCTCCTCCC

Amino acid composition and predicted MW of PCSK9

Full-length PCSK9 (precursor 74.286 kD, mature 57.273 kD)

MGTVSSRRSWWPLPLLLLLLLLLGPAGARAQEDEDGDYEELVLALRSEEDGLAEAPEHGT

TATFHRCAKDPWRLPGTYVVVLKEETHLSQSERTARRLQAQAARRGYLTKILHVFHGLLP

GFLVKMSGDLLELALKLPHVDYIEEDSSVFAQSIPWNLERITPPRYRADEYQPPDGGSLV

EVYLLDTSIQSDHREIEGRVMVTDFENVPEEDGTRFHRQASKCDSHGTHLAGVVSGRDAG

VAKGASMRSLRVLNCQGKGTVSGTLIGLEFIRKSQLVQPVGPLVVLLPLAGGYSRVLNAA

CQRLARAGVVLVTAAGNFRDDACLYSPASAPEVITVGATNAQDQPVTLGTLGTNFGRCVD

LFAPGEDIIGASSDCSTCFVSQSGTSQAAAHVAGIAAMMLSAEPELTLAELRQRLIHFSA

KDVINEAWFPEDQRVLTPNLVAALPPSTHGAGWQLFCRTVWSAHSGPTRMATAVARCAPD

EELLSCSSFSRSGKRRGERMEAQGGKLVCRAHNAFGGEGVYAIARCCLLPQANCSVHTAP

PAEASMGTRVHCHQQGHVLTGCSSHWEVEDLGTHKPPVLRPRGQPNQCVGHREASIHASC

CHAPGLECKVKEHGIPAPQEQVTVACEEGWTLTGCSALPGTSHVLGAYAVDNTCVVRSRD

VSTTGSTSEGAVTAVAICCRSRHLAQASQELQ

Exclusion of exon 2 (precursor 66.990 kD, mature 57.273 kD)

MGTVSSRRSWWPLPLLLLLLLLLGPAGARAQEDEDGDYEELVLALRSEEDGLAEAPEHGT

TATFHRCAKALKLPHVDYIEEDSSVFAQSIPWNLERITPPRYRADEYQPPDGGSLV

EVYLLDTSIQSDHREIEGRVMVTDFENVPEEDGTRFHRQASKCDSHGTHLAGVVSGRDAG

VAKGASMRSLRVLNCQGKGTVSGTLIGLEFIRKSQLVQPVGPLVVLLPLAGGYSRVLNAA

CQRLARAGVVLVTAAGNFRDDACLYSPASAPEVITVGATNAQDQPVTLGTLGTNFGRCVD

LFAPGEDIIGASSDCSTCFVSQSGTSQAAAHVAGIAAMMLSAEPELTLAELRQRLIHFSA

KDVINEAWFPEDQRVLTPNLVAALPPSTHGAGWQLFCRTVWSAHSGPTRMATAVARCAPD

EELLSCSSFSRSGKRRGERMEAQGGKLVCRAHNAFGGEGVYAIARCCLLPQANCSVHTAP

PAEASMGTRVHCHQQGHVLTGCSSHWEVEDLGTHKPPVLRPRGQPNQCVGHREASIHASC

CHAPGLECKVKEHGIPAPQEQVTVACEEGWTLTGCSALPGTSHVLGAYAVDNTCVVRSRD

VSTTGSTSEGAVTAVAICCRSRHLAQASQELQ

Exclusion of exon 8 (precursor 67.966 kD, mature 50.953 kD)

MGTVSSRRSWWPLPLLLLLLLLLGPAGARAQEDEDGDYEELVLALRSEEDGLAEAPEHGT

TATFHRCAKDPWRLPGTYVVVLKEETHLSQSERTARRLQAQAARRGYLTKILHVFHGLLP

GFLVKMSGDLLELALKLPHVDYIEEDSSVFAQSIPWNLERITPPRYRADEYQPPDGGSLV

EVYLLDTSIQSDHREIEGRVMVTDFENVPEEDGTRFHRQASKCDSHGTHLAGVVSGRDAG

VAKGASMRSLRVLNCQGKGTVSGTLIGLEFIRKSQLVQPVGPLVVLLPLAGGYSRVLNAA

CQRLARAGVVLVTAAGNFRDDACLYSPASAPEVITVGATNAQDQPVTLGTLGTNFGRCVD

LFAPGEDIIGASSDCSTCFVSQSGTSQAAAHVAGWQLFCRTVWSAHSGPTRMATAVARCAPD

EELLSCSSFSRSGKRRGERMEAQGGKLVCRAHNAFGGEGVYAIARCCLLPQANCSVHTAP

PAEASMGTRVHCHQQGHVLTGCSSHWEVEDLGTHKPPVLRPRGQPNQCVGHREASIHASC

CHAPGLECKVKEHGIPAPQEQVTVACEEGWTLTGCSALPGTSHVLGAYAVDNTCVVRSRD

VSTTGSTSEGAVTAVAICCRSRHLAQASQELQ

Exclusion of exon 9 (precursor 50.377 kD, mature 33.364 kD)

MGTVSSRRSWWPLPLLLLLLLLLGPAGARAQEDEDGDYEELVLALRSEEDGLAEAPEHGT

TATFHRCAKDPWRLPGTYVVVLKEETHLSQSERTARRLQAQAARRGYLTKILHVFHGLLP

GFLVKMSGDLLELALKLPHVDYIEEDSSVFAQSIPWNLERITPPRYRADEYQPPDGGSLV

EVYLLDTSIQSDHREIEGRVMVTDFENVPEEDGTRFHRQASKCDSHGTHLAGVVSGRDAG

VAKGASMRSLRVLNCQGKGTVSGTLIGLEFIRKSQLVQPVGPLVVLLPLAGGYSRVLNAA

CQRLARAGVVLVTAAGNFRDDACLYSPASAPEVITVGATNAQDQPVTLGTLGTNFGRCVD
LFAPGEDIIGASSDCSTCFVSQSGTSQAAAHVAGIAAMMLSAEPELTLAELRQRLIHFSA
KDVINEAWFPEDQRVLTPNLVAALPPSTHGAGPRGQAGLPGPQRFWG

Exclusion of exon 10 (precursor 56.210 kD, mature 39.197 kD)
MGTVSSRRSWWPLPLLLLLLLLLGPAGARAQEDEDGDYEELVLALRSEEDGLAEAPEHGT
TATFHRCAKDPWRLPGTYVVVLKEETHLSQSERTARRLQAQAARRGYLTKILHVFHGLLP
GFLVKMSGDLLELALKLPHVDYIEEDSSVFAQSIPWNLERITPPRYRADEYQPPDGGSLV
EVYLLDTSIQSDHREIEGRVMVTDFENVPEEDGTRFHRQASKCDSHGTHLAGVVSGRDAG
VAKGASMRSLRVLNCQGKGTVSGTLIGLEFIRKSQLVQPVGPLVVLLPLAGGYSRVLNAA
CQRLARAGVVLVTAAGNFRDDACLYSPASAPEVITVGATNAQDQPVTLGTLGTNFGRCVD
LFAPGEDIIGASSDCSTCFVSQSGTSQAAAHVAGIAAMMLSAEPELTLAELRQRLIHFSA
KDVINEAWFPEDQRVLTPNLVAALPPSTHGAGWQLFCRTVWSAHSGPTRMATAVARCAPD
EELLSCSSFSRSGKRRGERMEAAAPTGRWRTLAPTSRLC

Exclusion of exon 11 (cryptic splicing) (precursor 74.846 kD, 57.833 kD)
MGTVSSRRSWWPLPLLLLLLLLLGPAGARAQEDEDGDYEELVLALRSEEDGLAEAPEHGT
TATFHRCAKDPWRLPGTYVVVLKEETHLSQSERTARRLQAQAARRGYLTKILHVFHGLLP
GFLVKMSGDLLELALKLPHVDYIEEDSSVFAQSIPWNLERITPPRYRADEYQPPDGGSLV
EVYLLDTSIQSDHREIEGRVMVTDFENVPEEDGTRFHRQASKCDSHGTHLAGVVSGRDAG
VAKGASMRSLRVLNCQGKGTVSGTLIGLEFIRKSQLVQPVGPLVVLLPLAGGYSRVLNAA
CQRLARAGVVLVTAAGNFRDDACLYSPASAPEVITVGATNAQDQPVTLGTLGTNFGRCVD
LFAPGEDIIGASSDCSTCFVSQSGTSQAAAHVAGIAAMMLSAEPELTLAELRQRLIHFSA
KDVINEAWFPEDQRVLTPNLVAALPPSTHGAGWQLFCRTVWSAHSGPTRMATAVARCAPD
EELLSCSSFSRSGKRRGERMEAQGGKLVCRAHNAFGGEGVYAIARCCLLPQANCSVHTAP
PAEASMGTRVHCHQQGHVLTGCSSHWEVEDLGTHKPPVLRPRGQPNQCVGHREASIHAS
CCHAPGDRGLRGGLDPDWLQCPPWDLPRPGGLRRRQHVCSQEPGRQHYRQHQRRGRD
SRCHLLPEPAPGAGLPGAPVTAPSQDGCLGRVKGWG

Supplementary figures

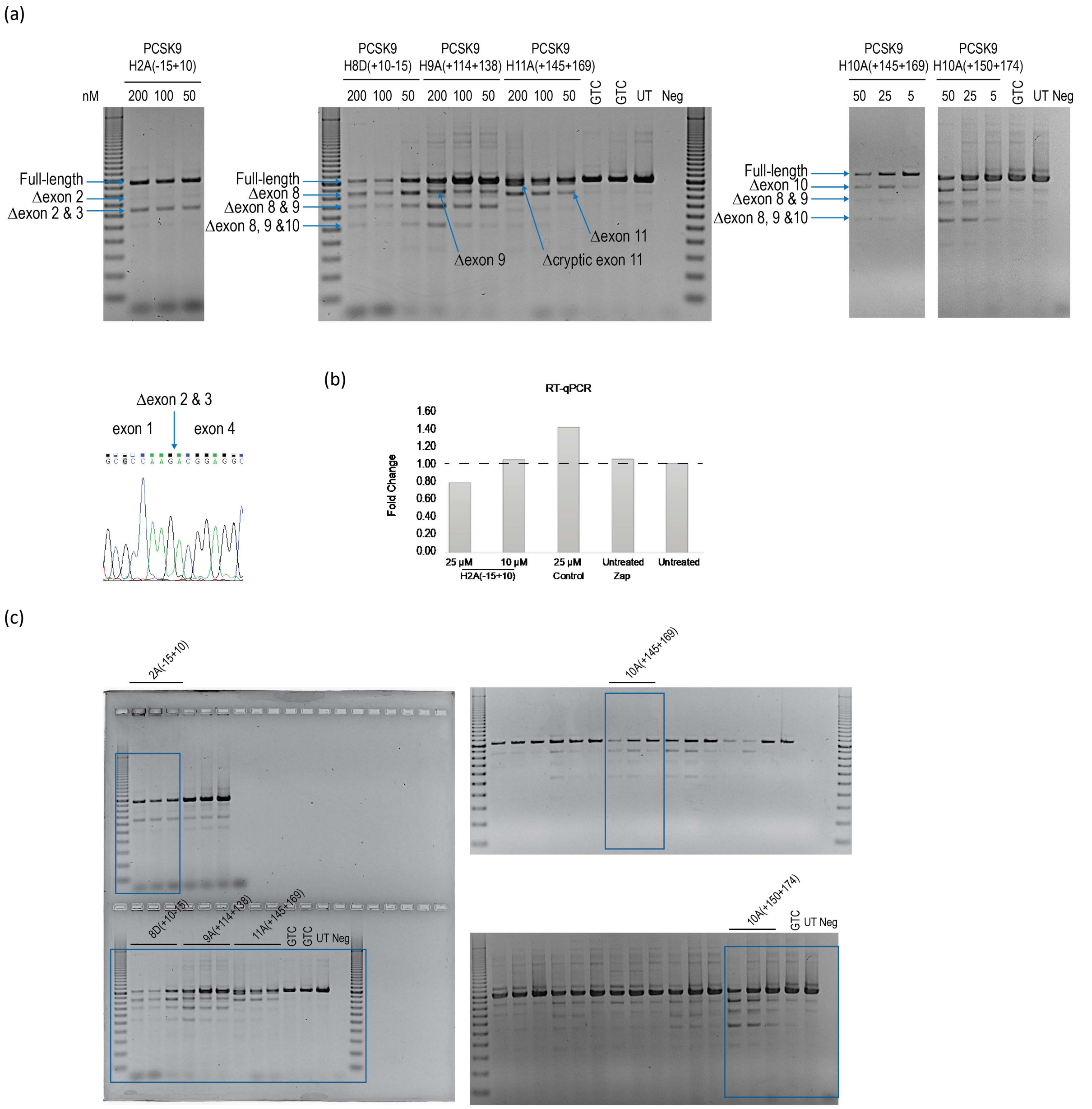

Supplementary figure 1. (a) RT-PCR analysis of *PCSK9* transcripts in Huh 7 cells after transfection with 2′OMe PS AOs targeting exon 2, 8, 9, 10 and 11. (b) RT-qPCR analysis of full-length PCSK9 after treatment with exon 2 targeting PMO. Crtl; samples treated with GeneTools control AO. UT; untreated. Neg; RT-PCR without template. (c) Original gels for panel (a).

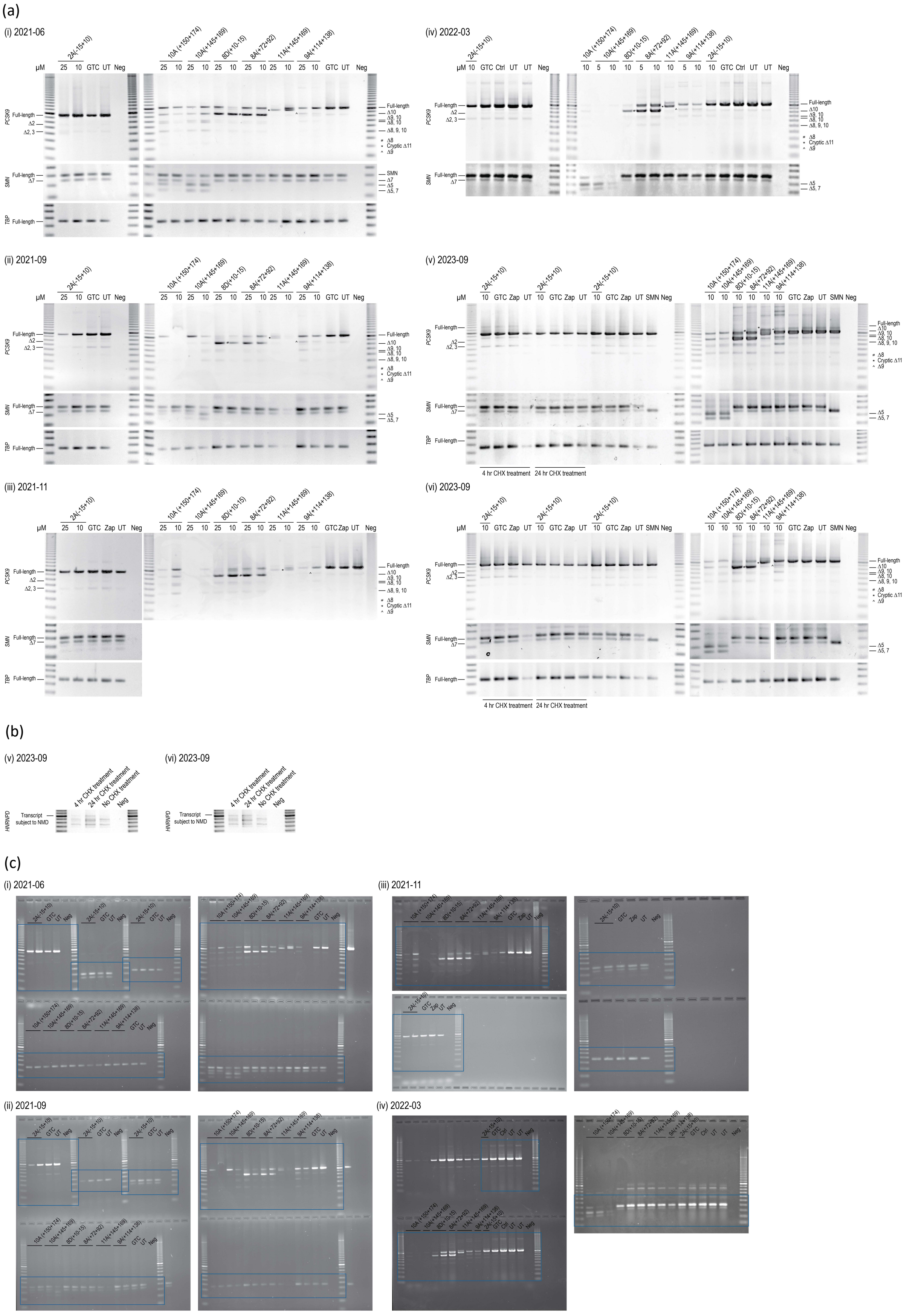

(a)
(i) 2021-06
(ii) 2021-09
(iii) 2021-11
(iv) 2022-03
(v) 2023-09
(vi) 2023-09
μM
2A(-15+10)
10A (+150+174)
10A(+145+169)
8D(+10-15)
8A(+72+92)
11A(+145+169)
9A(+114+138)
GTC
UT
Zap
Ctrl
SMN
Neg
PCSK9
SMN
TBP
Full-length
Δ2
Δ2, 3
Δ10
Δ9, 10
Δ8, 10
Δ8, 9, 10
Δ8
Cryptic Δ11
Δ9
Δ7
Δ5
Δ5, 7
4 hr CHX treatment
24 hr CHX treatment
(b)
(v) 2023-09
(vi) 2023-09
HNRNPD
Transcript subject to NMD
4 hr CHX treatment
24 hr CHX treatment
No CHX treatment
Neg
(c)
(i) 2021-06
(ii) 2021-09
(iii) 2021-11
(iv) 2022-03

(c) continued

(v) 2023-09

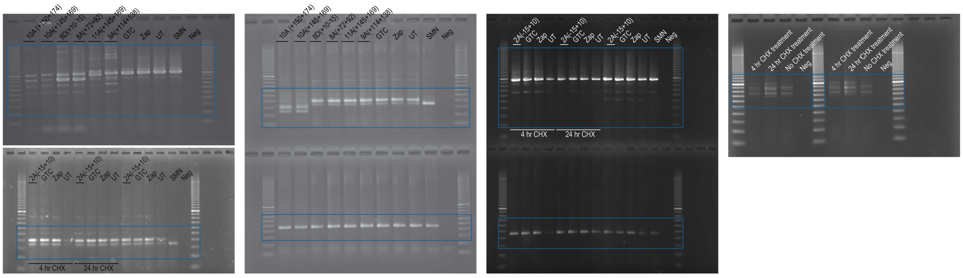

(vi) 2023-09

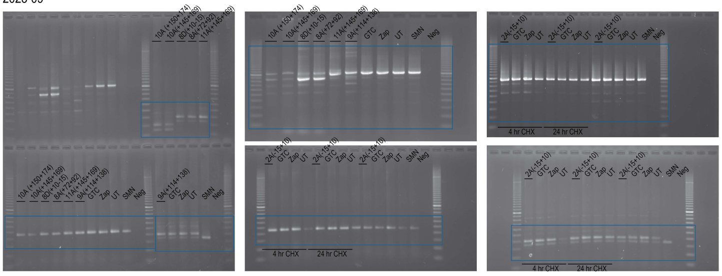

Supplementary figure 2. Dated replicates of phosphorodiamidate morpholino oligomer (PMO)-mediated exon skipping of proprotein convertase subtilisin kexin 9 (*PCSK9*) gene transcript. (a) Assessment of exon skipping from *PCSK9* transcripts in Huh-7 cells after treatment with PMO for three days using RT-PCR. Survival motor neuron (*SMN*) and TATA-box binding protein (*TBP*) transcripts were amplified as internal controls for RNA quality. Δ; removal of an exon, GTC; sample treated with Gene Tools control PMO, Zap; sample subject to Neon electroporation with no PMO, UT; untreated sample, Neg; RT-PCR without template added. (b) RT-PCR amplification of heterogeneous nuclear ribonucleoprotein D (*HNRNPD*) transcripts, as a positive control for inhibition of nonsense mediated decay (NMD) after cycloheximide (CHX) treatment. (c) Uncropped original gel images of panels (a) and (b).

(a)

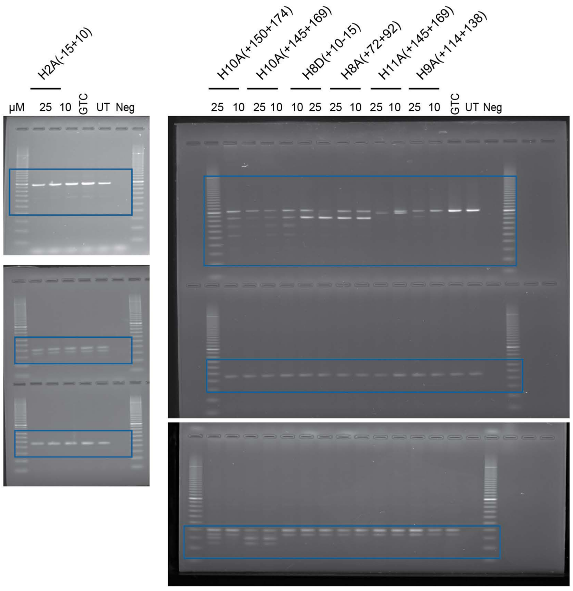

(b)

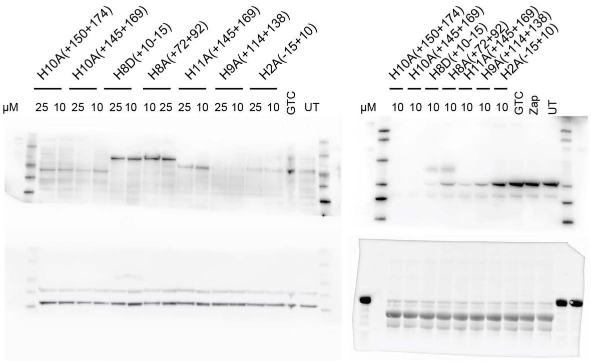

Supplementary figure 3. The uncropped original images for (a) Figure 1 and (b) Figure 2.

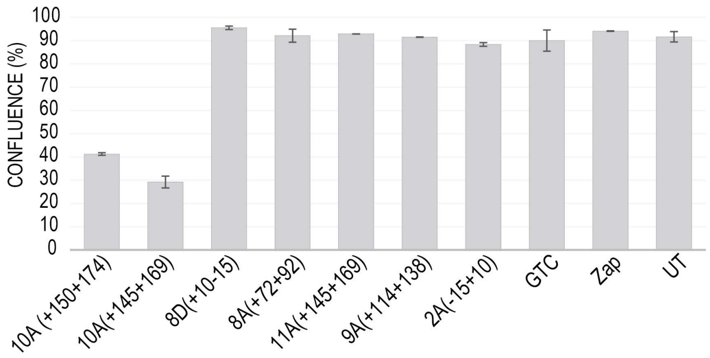

Supplementary figure 4. Assessment of confluence as a measure of cell death after treatment of Huh-7 cells with PCSK9 PMOs. Confluence was determined using the Incucyte® SX5 live-cell imaging system and provided software.

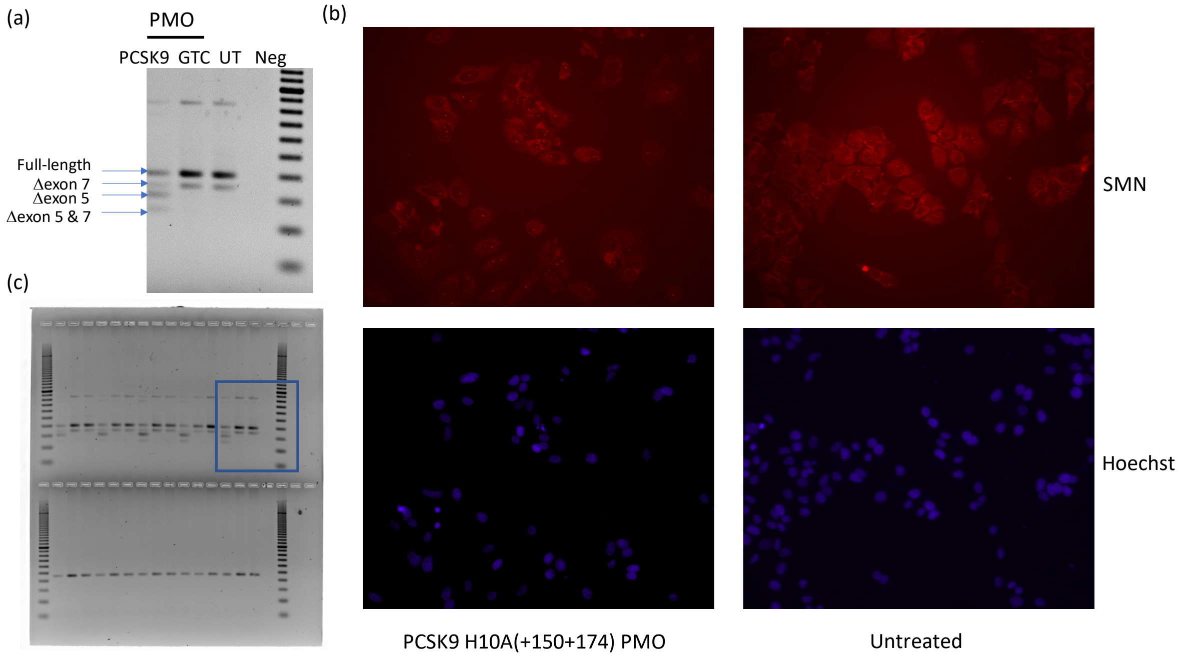

Supplementary figure 5. Assessment of gem formation after treatment with PCSK9 PMO H10A(+150+174). (a) RT-PCR analysis of *SMN* transcripts in Huh 7 cells after treating with PCSK9 PMO for 48 hours. GTC: sample treated with GTC PMO, UT; untreated. Neg; RT-PCR without template. (b) Analysis of gems after immunolabelling of SMN (red) and nuclei (blue). (c) Original gel for panel (a).

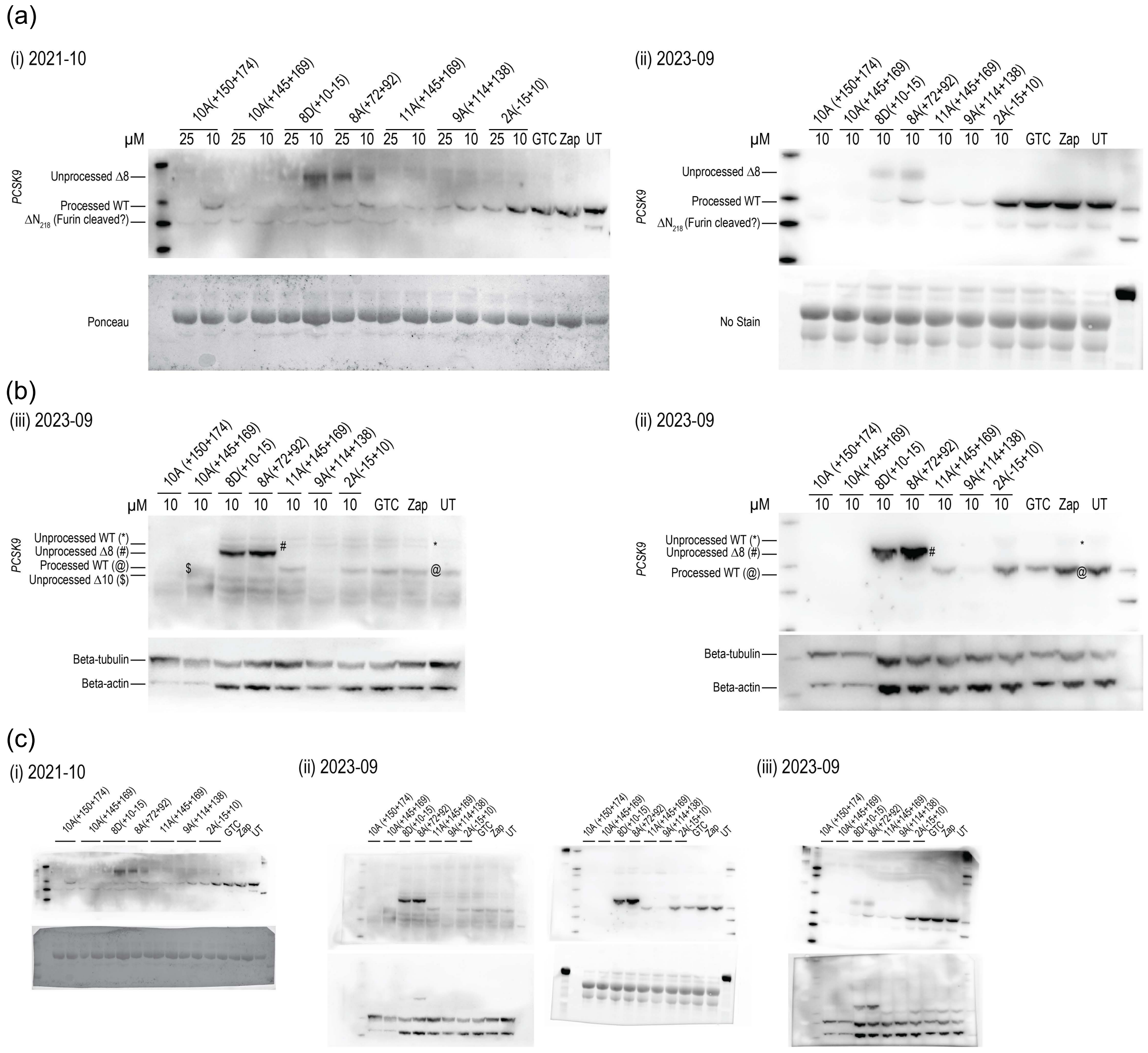

Supplementary figure 6. Dated replicates of PCSK9 protein isoform expression assessment in Huh-7 cells after treating with PMOs for three days. Western blot analysis of (a) secreted PCSK9 isoforms after targeted exon skipping and (b) intracellular PCSK9 protein isoforms. WT; wild-type, GTC; sample treated with Gene Tools control PMO, Zap; sample underwent Neon electroporation without any PMO, UT2; untreated sample. (c) Uncropped original images of panels (a) and (b).

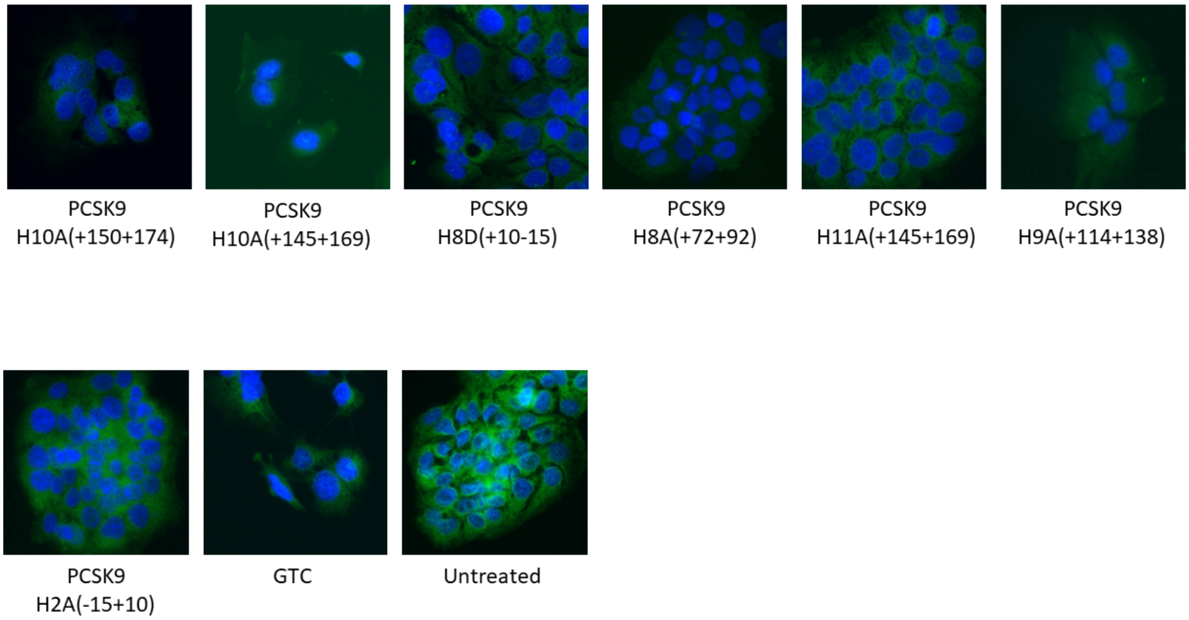

Supplementary figure 7. Analysis of the distribution of intracellular PCSK9 protein isoforms after treating with PMOs to induce exon skipping. The name of the PMO is shown below. GTC; GeneTools control. Green; PCSK9, blue; nuclei.

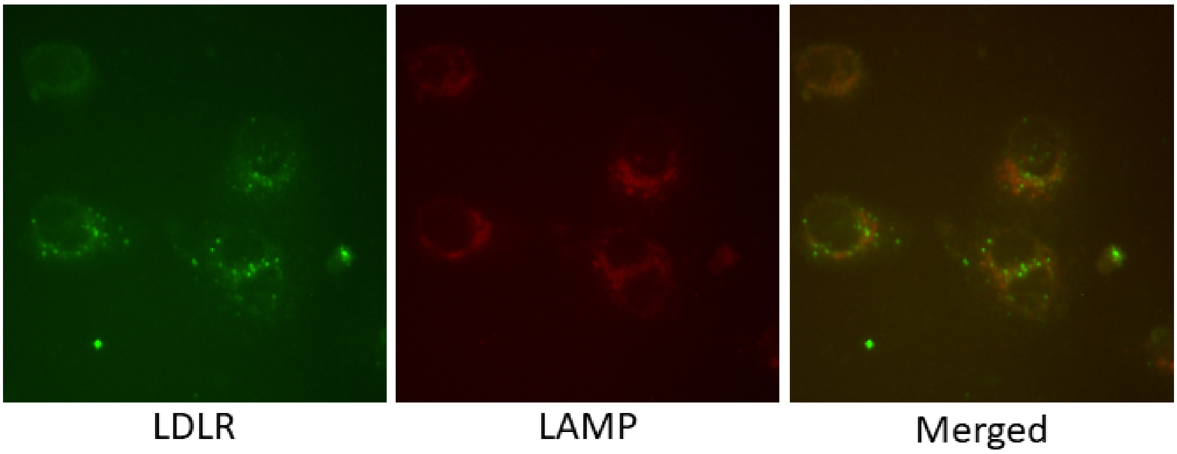

Supplementary figure 8. Analysis of LDLR and LAMP1 in Huh-7 cells treated with PMO H10A(+145+169) for three days.

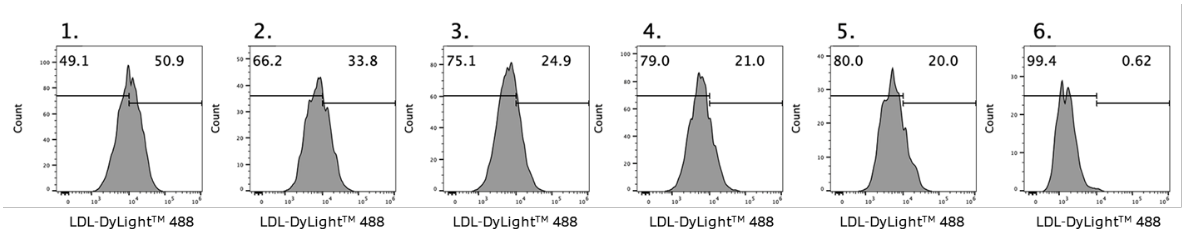

1. PCSK9 siRNA (10 nM)
2. PCSK9 siRNA (5 nM)
3. Negative control siRNA (10 nM)
4. Negative control siRNA (5 nM)
5. Untreated
6. Untreated with no LDL

Supplementary figure 9. The LDL uptake of Huh-7 cells after treatment with siRNA for four days.

Supplementary Table 1. Coordinates and sequences for 2′OMe PS AOs and PMOs

AO Names and Coordinates	2′OMe PS AOs	% Knockdown*	PMOs
PCSK9 H2A(-15+10)	UCCACGGAUCCUGGCCCCAUGCAAG	61.5	TCCACGGATCCTGGCCCCATGCAAG
PCSK9 H2A(+37+61)	GCGAGAGGUGGGUCUCCUCCUUCAG	22.8	
PCSK9 H2A(+75+99)	AGCCUGGGCCUGCAGGCGGCGGGCA	33.0	
PCSK9 H2A(+110+134)	ACAUGCAGGAUCUUGGUGAGGUAUC	7.7	
PCSK9 H2A(+158+182)	AGGUCGCCACUCAUCUUCACCAGGA	36.6	
PCSK9 H2D(+10-15)	AAAAGGGUGGCUCACCAGCUCCAGC	20.5	
PCSK9 H8A(-05+20)	CAGCAUCAUGGCUGCAAUGCCUGGU	26.6	
PCSK9 H8A(+68+92)	GAUGACAUCUUUGGCAGAGAAGUGG	16.1	
PCSK9 H8A(+72+92)	GAUGACAUCUUUGGCAGAGAA	Not tested #	GATGACATCTTTGGCAGAGAA
PCSK9 H8A(+100+124)	CCCGCUGGUCCUCAGGGAACCAGG	11.8	
PCSK9 H8D(+10-15)	UGCCAUCCUGCUUACCUGCCCCAUG	80.0	TGCCATCCTGCTTACCTGCCCCATG
PCSK9 H9A(-02+23)	AGUCCUGCAAAACAGCUGCCAACCU	5.7	
PCSK9 H9A(+30+54)	UCCGUGUAGGCCCCGAGUGUGCUGA	0.0	
PCSK9 H9A(+60+84)	CUGGGGCGCAGCGGGCGACGGCUGU	24.0	
PCSK9 H9A(+85+109)	CUGGAGCAGCUCAGCAGCUCCUCAU	3.3	
PCSK9 H9A(+114+138)	CGCCCCGCCGCUUCCCACUCCUGGA	50.0	CGCCCCGCCGCTTCCCACTCCTGGA
PCSK9 H10A(-15+10)	CCCCUUGGGCCUUAGAGUCAAAGAC	28.7	
PCSK9 H10A(+25+49)	CACCCCCAAAAGCGUUGUGGGCCCG	42.0	
PCSK9 H10A(+75+99)	GCAGUUGGCCUGGGGUAGCAGGCAG	63.3	
PCSK9 H10A(+70+94)	UGGCCUGGGGUAGCAGGCAGCACCU	9.1	
PCSK9 H10A(+80+104)	ACGCUGCAGUUGGCCUGGGGUAGCA	15.8	
PCSK9 H10A(+150+174)	GAGGACGUGGCCCUGUUGGUGGCAG	70.0	GAGGACGTGGCCCTGTTGGTGGCAG
PCSK9 H10A(+145+169)	CGUGGCCCUGUUGGUGGCAGUGGAC	64.2	CGTGGCCCTGTTGGTGGCAGTGGAC
PCSK9 H10D(+24-01)	CCUGUGAGGACGUGGCCCUGUUGGU	56.5	
PCSK9 H11A(+01+25)	UCCACCUCCCAGUGGGAGCUGCAGC	24.5	
PCSK9 H11A(+46+70)	UGACCUCGUGGCCUCAGCACAGGCG	5.4	
PCSK9 H11A(+71+95)	CCUGUGGCCCACGCACUGGUUGGGC	27.1	
PCSK9 H11A(+113+137)	CAGACCUGGGGCAUGGCAGCAGGAA	28.8	
PCSK9 H11A(+145+169)	GCCGGGAUUCCAUGCUCCUUGACUU	50.0	GCCGGGATTCCATGCTCCTTGACTT
PCSK9 H11D(+05-20)	GGCCUCACGGGCCUCUUCACCUGCU	35.9	
GTC (GeneTools control)	CCUCUUACCUCAGUUACAAUUUAUA		CCTCTTACCTCAGTTACAATTTATA

*% Full-length *PCSK9* transcript knockdown was calculated after treatment with 2′OMe PS AOs. # Not test as 2′OMe.
